# Investigating the Discoloration of Leaves of *Dioscorea polystachya* Using Developed Atomic Absorption Spectrometry Methods for Manganese and Molybdenum

**DOI:** 10.3390/molecules29163975

**Published:** 2024-08-22

**Authors:** David Krüger, Alexander Weng, Daniel Baecker

**Affiliations:** 1 Department of Pharmaceutical Biology, Institute of Pharmacy, Freie Universität Berlin, Königin-Luise-Straße 2+4, 14195 Berlin, Germany; d.krueger@fu-berlin.de (D.K.); alexander.weng@fu-berlin.de (A.W.); 2 Department of Pharmaceutical and Medicinal Chemistry, Institute of Pharmacy, Freie Universität Berlin, Königin-Luise-Straße 2+4, 14195 Berlin, Germany

**Keywords:** atomic absorption spectrometry, *Dioscoreaceae*, *Dioscorea polystachya*, extraction, graphite furnace, HR CS AAS, leaf extract, manganese, molybdenum, yam

## Abstract

The Chinese yam (*Dioscorea polystachya*, DP) is promising for the food and pharmaceutical industries due to its nutritional value and pharmaceutical potential. Its proper cultivation is therefore of interest. An insufficient supply of minerals necessary for plant growth can be manifested by discoloration of the leaves. In our earlier study, magnesium deficiency was excluded as a cause. As a follow-up, this work focused on manganese and molybdenum. To quantify both minerals in leaf extracts of DP, analytical methods based on atomic absorption spectrometry (AAS) using the graphite furnace sub-technique were devised. The development revealed that the quantification of manganese works best without using any of the investigated modifiers. The optimized pyrolysis and atomization temperatures were 1300 °C and 1800 °C, respectively. For the analysis of molybdenum, calcium proved to be advantageous as a modifier. The optimum temperatures were 1900 °C and 2800 °C, respectively. Both methods showed satisfactory linearity for analysis. Thus, they were applied to quantify extracts from normal and discolored leaves of DP concerning the two minerals. It was found that discolored leaves had higher manganese levels and a lower molybdenum content. With these results, a potential explanation for the discoloration could be found.

## 1. Introduction

The tuber of the Chinese yam (*Dioscorea polystachya*, DP) is an important foodstuff in Africa and Asia [1]. In addition, the plant has pharmaceutical potential as it is used in Traditional Chinese Medicine (TCM) against diabetes [2]. A large number of ingredients, some of which are still unknown, are responsible for the nutritional importance and medicinal effects of DP. Further structural elucidation of the unknown compounds in DP is auspicious because they may play a role as future lead structures for drug development. 

Optimal cultivation conditions are necessary for the plant to produce sufficient quantities of the desired nutrients and ingredients [3]. This also includes an adequate supply of minerals for the biochemical processes involved in the formation of primary and secondary metabolites. The deprivation of such minerals can manifest itself in phenotypical changes to the leaves [4].

In our previous study [5], a lack of magnesium was initially assumed for the visual discoloration of leaves of DP. However, the analyses unexpectedly revealed that the yellowish leaves had higher levels of magnesium. The reason for discoloration is therefore not considered a deficiency of magnesium.

As a follow-up to the previous publication, the current work takes up the challenge of finding a potential cause of the discoloration. The relevant minerals addressed were manganese and molybdenum. Both (in)directly participate in the synthesis of chlorophyll [6,7]. Appropriate analytical methods are required for the quantification of these two elements in leaf extracts.

Atomic absorption spectrometry (AAS) is an established technique for the analysis of metals. Advantages are its versatility, low effort regarding sample preparation, and its sensitive performance [8]. Compared to inductively coupled plasma mass spectrometry (ICP MS) as an alternative technique, AAS is less expensive [9]. Therefore, the aim of this work was to develop two AAS-based methods, one for manganese and one for molybdenum. In particular, the AAS sub-technique was used, in which atomization takes place electrothermally in a graphite furnace (ET AAS/GF AAS).

The optimization of the methods was aimed especially at the essential temperatures of the pyrolysis and atomization operation steps. Moreover, the absorption signals obtained can be augmented by the use of suitable modifiers. Therefore, a series of such additives was also investigated. Finally, the two analytical methods were practically applied to the analysis of extracts prepared from normal and discolored leaves of DP. The objective was to determine whether there is a possible connection between the manganese and molybdenum content and the discoloration of the DP leaves.

## 2. Results and Discussion

### 2.1. Selection of the Wavelengths

**Figure 1:**
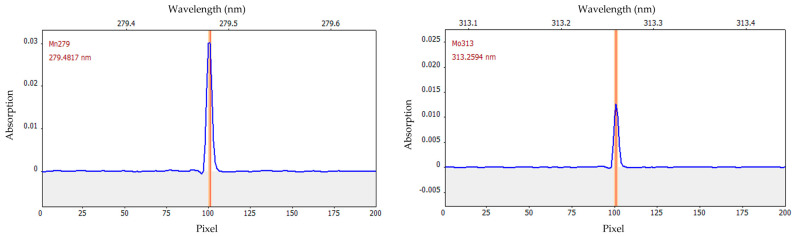
Wavelength-resolved absorption spectrum (blue) of manganese (Mn, (**left**)) and molybdenum (Mo, (**right**)). The red line indicates the wavelength of manganese (λ = 279.4817 nm) and molybdenum (λ = 313.2594 nm), respectively. The measurement of the integrated signal over three pixels is indicated by the orange bar. The spectra shown were recorded during the quantification of the calibration standards of 6 μg L^−1^ for manganese and 100 μg L^−1^ for molybdenum.

AAS belongs to the spectroscopic analytical techniques. The choice of the wavelengths plays a crucial role and depends on the analyte. For both manganese and molybdenum, the primary line was selected for quantification. It is λ = 279.4817 nm for manganese and λ = 313.2594 nm for molybdenum. In each case, this is the wavelength with 100% sensitivity, thus termed the primary line. The wavelength-resolved absorption spectra (Figure 1) exhibited no further lines in close vicinity of the isolated primary lines. Therefore, interferences due to the overlapping of lines can be excluded.

### 2.2. Optimization of the Time–Temperature Program for Manganese

The various sub-techniques of AAS differ in the mode of atomization. In GF AAS, this is achieved electrothermally in a graphite tube. The latter is heated transversely in a graphite furnace. Hence, the graphite furnace program (time–temperature program) plays an essential role in GF AAS. It generally consists of four major steps, i.e., drying, pyrolysis, atomization (including the actual measurement), and cleaning [10]. Each of these steps requires appropriate conditions with respect to temperature for instance.

Therefore, a graphite furnace program was developed for the determination of manganese and molybdenum, respectively. Four extracts were prepared from different leaves of DP. These extracts were diluted with water at a ratio of 1:10^6^ and 1:10 for the analysis of manganese and molybdenum, respectively. The approach of utilizing leaf extracts for the optimization procedure made it possible to consider potential matrix effects with regard to the subsequent analysis of the biological samples. This is particularly important for the operation step of pyrolysis, during which accompanying compounds are removed.

The procedure described by Welz was used to optimize the pyrolysis and atomization temperatures [11]. In the first approach, the pyrolysis temperature is kept constant and the atomization temperature is increased in steps of 100 °C (manganese: from 1400 °C to 2300 °C; molybdenum: from 2000 °C to 2800 °C). The ideal atomization temperature is the lowest temperature at which the highest absorption signal is achieved. Accordingly, to optimize the pyrolysis temperature at the fixed atomization temperature, a gradual rise (steps of 100 °C; manganese: from 900 °C to 1700 °C; molybdenum: from 1400 °C to 2000 °C) is carried out. The highest temperature with the strongest absorption signal is optimal. As part of this optimization process, modifiers can be used to further improve performance [12]. Ideally, they enhance the absorption signal and allow the steps to be carried out at more favorable temperatures.

Very common chemical modifiers are, for example, nitric acid [13], as well as the universal modifiers magnesium and palladium [14]. Since the investigated extracts are based on nitric acid, this was not analyzed specifically as a modifier. It was found in our previous study that the leaves of DP have a high content of magnesium [5]. For this reason, magnesium was not taken into account as an additional modifier. However, palladium (palladium nitrate) was used as a modifier, inspired by an overview of earlier AAS measurements [15]. Moreover, the addition of reducing agents such as tartaric acid or ascorbic acid has previously been shown to be efficient in obtaining palladium in its elemental form [16,17]. The metal proved to be successful in eliminating interferences [16]. Therefore, the combinations of palladium with tartaric acid and ascorbic acid, respectively, were examined. Finally, due to encouraging results in a previous study [18], calcium was also investigated as a modifier.

**Figure 2:**
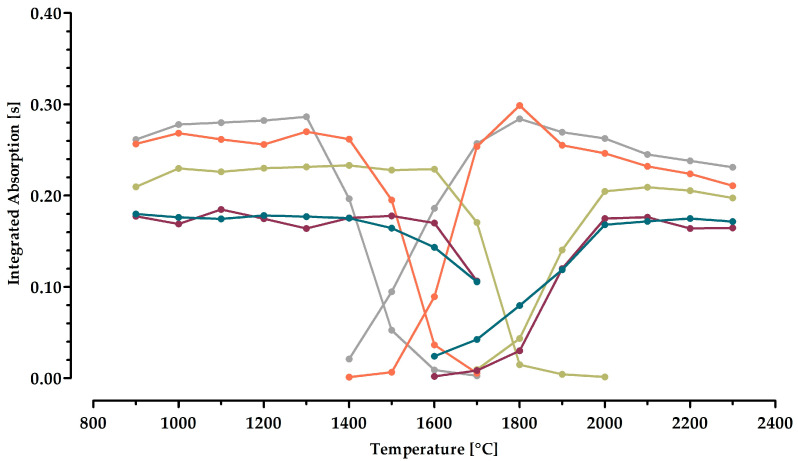
Pyrolysis (**left**) and atomization (**right**) curves for the assessment of manganese (λ = 279.4817 nm). A volume of 10 µL of diluted leaf extract was injected into the graphite furnace for each measurement. The curves represent the cases of using no additive (⬤) or adding the chemical modifiers palladium (⬤), palladium and tartaric acid (⬤), palladium and ascorbic acid (⬤), as well as calcium (⬤). The data represent the mean of four different extracts with three injections each. The error bars were omitted to allow better readability.

The results of optimizing the pyrolysis and atomization temperatures for the graphite furnace program of manganese are shown in Figure 2.

Following the Welz method without using any chemical modifier resulted in the highest signal of the integrated absorption (0.29 s) at a pyrolysis and atomization temperature of 1300 °C and 1800 °C, respectively. The temperature of the pyrolysis is in good agreement with studies reported previously on the determination of manganese in biological samples [16,19]. The temperature optimized for the atomization is lower in this work than in the earlier investigations. This is advantageous in terms of the lifetime of the furnace and the tubes.

When using the universal modifier palladium, the strongest signal (0.23 s) was obtained at a pyrolysis temperature of 1400 °C. Generally, pyrolysis temperatures as high as possible are advantageous to allow for more extensive removal of accompanying compounds. However, in this case, the signal was inferior to that of using no modifier, which is not favorable. As the optimized atomization temperature was 2000 °C (integrated absorption: 0.21 s) and thus higher than preferred, and since the signal further decreased slightly, palladium was withdrawn as a suitable modifier.

Reducing palladium to its metallic form using either tartaric acid or ascorbic acid was investigated, too. Both approaches resulted in similar curves. Applying tartaric acid in addition to palladium gave the highest signal (0.18 s) at 1100 °C and 2100 °C, respectively. In the case of ascorbic acid, the pyrolysis and atomization temperatures were 1200 °C and 2200 °C with identical absorption values (0.18 s). A combination of palladium with the reducing agents is not recommended.

Finally, calcium was investigated as a potential modifier. Its performance is comparable to the scenario when adding no modifier. The optimized temperatures were 1300 °C and 1800 °C and, thus, are the same as without a modifier. The signals (0.27 s and 0.30 s) were only marginally different. These negligible differences were considered not to justify the application of calcium as an additional modifier. Analytical methods should be as simple as possible. The omission of a modifier does not cause additional injection steps, makes the analysis a little less time- and cost-consuming, and does not harbor the risk of potential contamination. Therefore, it was decided in the current study not to apply any modifier when quantifying manganese in the leaf extracts of DP. Thus, the optimized pyrolysis and atomization temperatures are 1300 °C and 1800 °C, respectively. These are in accordance with previous GF AAS-based analyses of manganese [20].

The conditions of the drying step also play an important role in the time–temperature program. It must be ensured that, for example, boiling distortion potentially leading to loss of analyte is avoided through uniform drying. In this study, the same drying steps were used that were already optimized in our previous work [5]. In summary, the developed graphite furnace program is reported in Table 1.

### 2.3. Optimization of the Time–Temperature Program for Molybdenum

**Figure 3:**
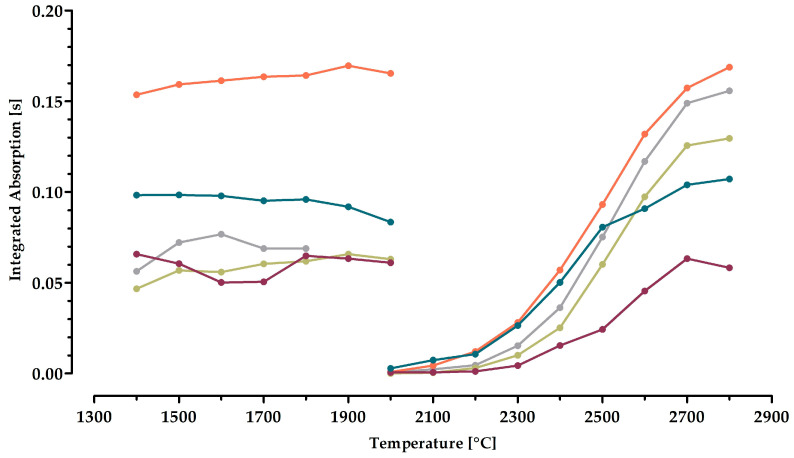
Pyrolysis (**left**) and atomization (**right**) curves for the assessment of molybdenum (λ = 313.2594 nm). A volume of 10 µL of diluted leaf extract was injected into the graphite furnace for each measurement. The curves represent the cases of using no additive (⬤) or adding the chemical modifiers palladium (⬤), palladium and tartaric acid (⬤), palladium and ascorbic acid (⬤), as well as calcium (⬤). The data represent the mean of four different extracts with two injections each. The error bars were omitted to allow better readability.

The curves of optimizing the temperatures for the quantification of molybdenum showed a different pattern (Figure 3).

Investigating of the temperatures without any chemical modifier gave the highest signal of the integrated absorption (0.08 s) at the pyrolysis temperature of 1600 °C. Unexpectedly, the signal was twice as high (0.16 s) when optimizing the atomization temperature. The latter amounted to 2800 °C. The noticeably low absorption values when measuring the same extracts regarding molybdenum compared to manganese indicates a poorer content of molybdenum in the leaf extracts. This has, in general, also been reported for other plants before [21]. However, the use of chemical modifiers can help enhance the absorption values.

Palladium, a universal modifier in AAS, was investigated. The pyrolysis curve revealed no increase in the absorption signal (0.07 s). Its use was accompanied by a higher temperature of 1900 °C. The ideal atomization temperature remained unchanged at 2800 °C, but gave a lower absorption (0.13 s). Therefore, palladium itself was not considered to be a suitable additive in the current analysis.

The influence of reducing palladium with tartaric acid and ascorbic acid, respectively, was studied as well. Using tartaric acid gave the temperatures of 1800 °C and 2700 °C. Although the slight decline in the atomization temperature upon combining palladium with tartaric acid would be favorable, e.g., with respect to the lifetime of the graphite tubes, the signal (0.06 s) was once again a little weaker than without any modifier. Palladium and ascorbic acid showed a somewhat better behavior. The pyrolysis and atomization temperatures were 1800 °C (0.10 s) and 2800 °C (0.11 s), but still did not really exceed the case of not applying a modifier. This is why these two approaches were also rejected.

The investigation of calcium led to an augmentation of the absorption signal (0.17 s). At this, the optimized pyrolysis and atomization temperatures were 1900 °C and 2800 °C, respectively. Based on this performance, it was decided to use calcium as a modifier for the analysis of molybdenum. The final time–temperature program is summarized in Table 2.

The atomization temperature of 2800 °C indeed seems to be quite high, but is not uncommon for the analysis of molybdenum with GF AAS [22]. This is due to the relatively high melting and boiling points of molybdenum [23]. The atomization temperatures of previous analyses, in which different plant materials and tissues were sampled, were in a similar range [24,25,26].

### 2.4. Analytical Parameters of the Manganese Method

The developed GF AAS method to determine manganese was characterized regarding the figures of merit (Table 3). A manganese standard solution for AAS was used to prepare the calibration standards by dilution with ultrapure water. The calibration curve covered ten standards (2–20 µg L^−1^) and one blank consisting of ultrapure water only. The squared correlation coefficient R^2^ = 0.9995 documented satisfactory linearity for quantification. The characteristic mass (m_0_), defining the mass of the analyte causing an integrated absorption of 0.0044 s [5], was assessed as mean (±standard deviation) from all the calibration standards. It was 0.67 pg and exceeded the values (1.42–4.74 pg) previously reported in the analysis of botanical samples [16], and thus documents the competitiveness of the method developed here.

The limit of detection (LOD) and the limit of quantification (LOQ) were determined as previously described [5]. The LOD for the quantification of manganese was 0.67 µg L^−1^ and the LOQ amounted to 1.83 µg L^−1^. For the determination of the recovery, a solution containing 2 µg L^−1^ of manganese was prepared from manganese(II) sulfate by diluting it with ultrapure water. The latter concentration was selected as it just reached the LOQ. The recovery was 98.5%, and the precision had a value of 1.9% (three measurements). In conclusion, these analytical figures approve a reliable quantification of manganese in the diluted extracts of leaves of DP.

### 2.5. Analytical Parameters of the Molybdenum Method

It is commonly known that the analysis of molybdenum is very challenging. This is due to the potential of molybdenum to generate carbides with the graphite surface of the tube. These possible carbides are thermally stable and hardly volatile [27]. Using the maximum possible temperatures has been suggested to address this issue, even if this is at the expense of the lifetime of the graphite tubes [28]. The complexity of analyzing molybdenum is also reflected in the analytical parameters of the method developed in this study. These are a little weaker (Table 4), but still within the range to enable quantification. From 10 to 100 µg L^−1^, the calibration (ten standards and one blank) showed linearity given the correlation coefficient of R^2^ = 0.9909. The characteristic mass amounted to 21.4 pg. The LOD and the LOQ were 9.94 µg L^−1^ and 37.64 µg L^−1^, respectively. An aqueous solution of molybdic acid (40 µg L^−1^) with a concentration close to the LOQ was prepared to assess the recovery and precision of the method. They were 100.7% and 3.8%, respectively.

### 2.6. Quantification of the Extracts Regarding Manganese and Molybdenum

The optimization of the parameters mentioned above served for the development of GF AAS-based methods. These were used to quantify the contents of manganese and molybdenum in leaf extracts of DP. This was of interest in order to obtain an insight into whether the two minerals could be involved in the discoloration of the leaves of DP. Our previous study already indicated that lack of magnesium is not the reason for the yellowish color of the leaves. Unexpectedly, the discolored leaves had a higher magnesium content than the normal leaves [5].

**Figure 4:**
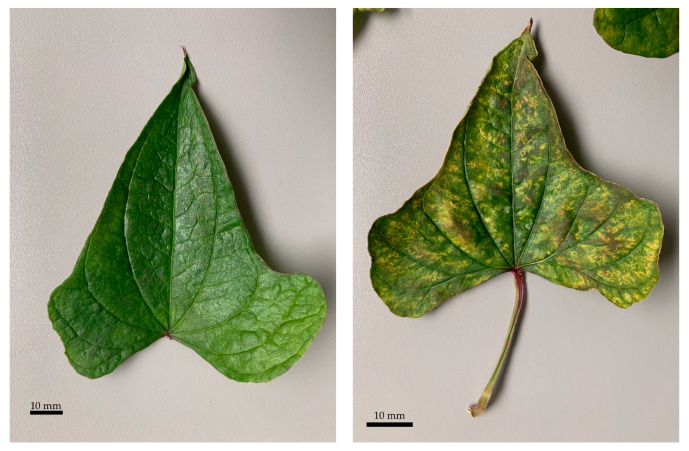
Exemplary illustration of a normal-colored leaf (**left**) and a discolored leaf (**right**) of *Dioscorea polystachya*. In addition to the yellowing of the deficient leaves, these also exhibit brownish areas.

Green leaves without phenotypically visible color changes were defined as normal leaves (NL). In contrast, discolored leaves (DL) exhibited visually apparent chlorosis (Figure 4). All four extracts (NL-1 to NL-4 and DL-1 to DL-4) were prepared from a random selection of normal and discolored leaves. These were quantified for manganese and molybdenum using the designed GF AAS methods. Under consideration of the mass of leaves that were employed to generate the analyzed extracts, the content of mineral is expressed as the mass of mineral (mass in mg) related to the mass of leaves (mass in kg). The results of the quantification are listed in Table 5.

The four extracts (1–4) made of normal leaves had a mean manganese content of 30.85 mg kg^−1^. In contrast, the discolored leaves exhibited an approximately 20% higher level (37.27 mg kg^−1^). This difference is statistically not significant. However, this might be due to the generally increased standard deviation for biological samples and the small sample size (n = 4). The latter can be considered as a limitation of the present study. It could be remedied by a larger number of samples. Due to the shortage of plant material, this was not possible in the current study.

Investigating the extracts concerning the molybdenum content gave a mean of 18.41 mg kg^−1^ in the normal-colored leaves and merely 15.87 mg kg^−1^ in the deficient leaves. Therefore, the regular leaves have a 16% greater level of molybdenum compared to the discolored ones. Again, the difference is statistically not significant, but might be significant when measuring more samples.

The discrepancy in the single values obtained within samples 1–4 of each of the two groups (NL and DL) seems somewhat striking. Several explanations could be considered. A different composition of the soil and thus varying degrees of mineral uptake can be virtually ruled out (even though the soil of each plant was not analyzed). The plants grew close to each other and all received the same anthropogenic treatment. Nonetheless, additional non-visible influences such as infestations of insects and fungi or damage of the roots (e.g., caused by rodents) can lead to chlorosis [29]. At this, the content of the two minerals would not play a primary role.

Chlorosis is also a hallmark of the natural senescence process. For example, chlorosis due to a deficiency of magnesium appears preferentially in old leaves until it extends to the young ones [30,31]. Lack of magnesium is not present in the current investigation. However, it is mainly the matured leaves that are affected by yellowing with additional brown discoloration due to excessive manganese [29]. For chlorosis induced by molybdenum deficiency, there are also reports that first the older and then the younger leaves are involved [32,33]. It might be possible that the analyzed leaves (1–4) were unconscious of different aging processes at the time the samples were harvested and therefore had varying levels of the two minerals, but yet showed discoloration. Unfortunately, it was not possible to trace whether the yellow leaf samples came from the young shoots or the older ones. However, this is not a significant issue, as the entire plant was affected at the time of sample collection.

An important aspect that could explain the discrepancies in manganese content is that chlorosis can be caused not only by excessive manganese, but also by its deficiency [6,34]. The question of the thresholds for normal mineral content, deficiency, or excess cannot be answered in a simple way. Manganese levels of 20–40 mg kg^−1^ are considered to be regular [35]. For the accumulation of manganese in plants, following symptoms of toxicity such as chlorosis, a range from 20 up to 500 mg kg^−1^ is reported [36]. In our case, the mean values are just on the borderline between vital and abnormal. However, it is the leaves that often show increased content in manganese [37,38]. Yet, it is noteworthy that there are variations between the limits of tolerance for manganese in various plants on the one hand, and on the other, the level can differ even in genotypes of the same plant species [36]. DP could be a species that is naturally more susceptible to manganese.

It is challenging to draw any conclusions about the thresholds of manganese for DP. What can be deduced, however, when looking at the mean values obtained, is that discolored leaves had an elevated content of manganese and less molybdenum compared to the normal-colored leaves. The level at which manganese is toxic to plants cannot be said in a generalized term, as it depends on the species [39]. However, if manganese toxicity is evident, this manifests itself in chlorosis of the leaves [34,40]. This has been described and reviewed in recent studies for a variety of plants [34,36,41,42,43,44].

There is a correlation between high amounts of manganese, a decrease in chlorophyll (causing fading of the leaves), and changes in the pattern of its precursors. For example, an accumulation of the magnesium protoporphyrin IX monomethyl ester has been reported in the literature [45]. This indicates that chlorosis is not necessarily related to a reduced magnesium content. This was the case here because the discolored leaves had higher levels of magnesium compared to the normal ones.

Manganese-induced chlorosis also applies to the leaves of DP, since the yellowish leaves had a higher manganese content (Figure 4 and Table 5). In our case, this was accompanied by a lower molybdenum content. This observation is likewise consistent, as increased manganese can affect the uptake of molybdenum [46].

A lack of molybdenum results in the enrichment of nitrates, which, however, cannot be reduced to amines. This process requires so-called molybdoenzymes, i.e., molybdenum-dependent nitrate reductases. Since amines are crucial for the synthesis of amino acids and these, in turn, again, for the generation of chlorophyll, a deficiency of molybdenum can lead to discoloration as well [7,47]. Some sulfite oxidases belong to the class of molybdoenzymes. These enzymes are involved in the metabolism of sulfur-containing molecules. Furthermore, sulfite oxidase catalyzes the oxidation and thus the detoxification of sulfite to sulfate [48]. The former leads to chlorophyll degradation, depending on the availability of sulfur dioxide in the atmosphere. As a result of chlorophyll decomposition, the leaves show chlorosis [49]. However, the respective threshold for toxic effects again depends on the plant variety. If one or both of the aforementioned enzymes are deficient in plants, phenotypic changes such as discolored leaves occur [50].

In addition, manganese toxicity is associated with brownish-stained areas on the leaves, which can be attributed to the accumulation of manganese in terms of manganese oxide [39,51]. The discolored leaves examined were also found to have brown spots (Figure 4), thus confirming the hypothesis of manganese toxicity.

Manganese toxicity results from an excessive uptake of the mineral mainly from acidic soil [52]. In general, the nature of the soil has an influence on the synthesis of ingredients in the plant (e.g., secondary metabolites) as well as on the mineralization [53]. However, investigating this was not part of the current work, but could provide further information in coming studies.

It should also be mentioned that a deficiency of other minerals (e.g., boron, copper, iron, phosphorus, potassium, sulfur, zinc) can be associated with the symptoms of chlorosis [29]. A more comprehensive screening could therefore provide even more insights in upcoming studies. Nevertheless, light was shed on the fact that manganese toxicity and molybdenum deficiency might be one explanation for the discoloration of the leaves of DP. This knowledge could be implemented in the future to optimize the cultivation of DP and thus intensify research regarding the clarification of its secondary metabolites. The latter may be of interest for drug development, especially since DP is already used in TCM.

## 3. Materials and Methods

### 3.1. Chemicals

All the chemicals, modifiers, and solvents employed in the experiments were obtained from Merck (Darmstadt, Germany), Carl Roth (Karlsruhe, Germany), and Sigma Chemical Company (St. Louis, MO, USA). They were used without additional purification. In particular, the commercially available manganese standard solution for AAS (1 mg mL^−1^ Mn in 2 to 5% HNO_3_, Thermo Fisher Scientific, Fair Lawn, NJ, USA) and the molybdenum AAS standard solution (Specpure^®^, 1000 µg mL^−1^ Mo, Thermo Fisher Scientific, Kandel, Germany) were utilized for the preparation of the calibration standards. For this purpose and for the dilution of the samples, ultrapure water (conductance 0.055 µS cm^−1^; Siemens LaboStar, Gunzburg, Germany) was used. The chemical modifiers used per injection were 5 µL of palladium (0.1% solution in 10% HNO_3_, i.e., 5 µg), 5 µL of palladium (0.1% solution in 10% HNO_3_, i.e., 5 µg) and 2 µL of tartaric acid (40 g L^−1^), 5 µL of palladium (0.1% solution in 10% HNO_3_, i.e., 5 µg) and 2 µL of ascorbic acid (10 g L^−1^), as well as 5 µL of calcium (1 g L^−1^ solution in 2% (*w*/*w*) HNO_3_, i.e., 5 µg). Solutions made of manganese (II) sulfate monohydrate (reagent Ph. Eur. for analysis, AppliChem, Darmstadt, Germany) and molybdic acid (Thermo Fisher Scientific, Kandel, Germany) were prepared to determine the recovery. Nitric acid 65% (*w*/*v*) (Merck, Darmstadt, Germany) was taken for the extraction, which was diluted with the ultrapure water to give 12% nitric acid (*w*/*v*).

### 3.2. Plant Collection and Extraction

The Andreashof (Überlingen, Germany) was the supplier of the DP leaves. It is a certified association for cultivating Chinese yam. The DP plants (height: 3–4 m), from which the leaves used in this study were obtained by hand-picking at eye level, were cultivated close to each other on the same soil. The leaves analyzed were taken from different plants grown side by side in one ditch habitat over an area of around 50 m^2^. The normal leaves and those with discoloration were collected at the same time (age of plants: 5–6 months). An internal and external quality control of the cultivation is guaranteed by the Andreashof and Demeter (Darmstadt, Germany), respectively.

Acidic extracts were made of dried leaves (normal and discolored leaves). For this purpose, the leaves were crushed into a powder through a mortar and then extracted. One part of the powder was dissolved in ten parts of nitric acid (12%, *w*/*v*). The flasks were lidded with a watch glass and extracted in a water bath (100 °C) for 2 h. Then, the hot suspensions were filtered through round filters (grade 595, diameter: 70 mm; Schleicher and Schuell, Dasse, Germany). Until being analyzed further, the extracts were kept in roll rim snap lid jars (VWR International, Darmstadt, Germany) at room temperature and under light protection.

For quantifying manganese and molybdenum, the extracts were diluted with ultrapure water at a ratio of 1:10^6^ and 1:10, respectively.

### 3.3. Atomic Absorption Spectrometer and Procedure

All AAS measurements were performed with a high-resolution continuum-source atomic absorption spectrometer contrAA 700 (Analytik Jena, Jena, Germany). The graphite furnace (GF) AAS sub-technique was followed. The software Aspect CS version 2.3.1.0 (Analytik Jena) was used to run the spectrometer. The purging and protecting gas of the spectrometer was 99.999% argon (Alphagaz, Air Liquide, Düsseldorf, Germany). The samples to be analyzed were stored in 1.5 mL polystyrole vials (Sarstedt, Nurmbrecht, Germany). Injection of the samples into pyrolytically coated standard graphite tubes was performed with the MPE 60 graphite furnace autosampler (Analytik Jena). A xenon short-arc lamp provided continuous light (wavelengths of 185–900 nm). High resolution was achieved by a double monochromator with a quartz prism pre-monochromator and an echelle grating monochromator (slit width: 50 μm width, 1 mm height), and a linear charge-coupled device (CCD) array detector. The integrated absorption (primary absorption lines, three pixels) was adduced for the quantification. In particular, the wavelengths were λ = 279.4817 nm for manganese and λ = 313.2594 nm for molybdenum. The optimized graphite furnace programs are given above (Table 1 and Table 2). The measurements were based on the mean integrated absorption of triplicate injections when assessing manganese. Due to the very high temperatures involved in the analysis of molybdenum, only two injections were carried out to. This should save the lifetime of the graphite furnace and tubes.

### 3.4. Statistical Analysis

The unpaired *t* test (GraphPad Prism 5.0, San Diego, CA, USA) was used to analyze the significance (*p* < 0.05) of differences between the mineral content of normal and discolored leaves.

## 4. Conclusions

The Chinese yam (*Dioscorea polystachya*, DP) is of social interest due to its nutritional and pharmaceutical value. Although the active substances of DP that induce biological effects have not yet been completely elucidated, the plant has great potential to comprise molecules that may serve as lead structures for future drug development. Therefore, ideal cultivation conditions are desirable. In contrast, insufficient growth or inadequate supply of essential minerals can be indicated by a change in the color of the leaves. In our previous publication, a lack of magnesium (the metal of chlorophyll) was unexpectedly ruled out as a cause of discoloration. In this study, the two minerals manganese and molybdenum were addressed, which are also somehow involved in the synthesis of chlorophyll.

GF AAS-based methods were developed. The main focus was on the optimization of the graphite furnace program. None of the investigated modifiers proved to be really useful for the quantification of manganese. Thus, the omission of an additive made the method simpler. The optimized pyrolysis and atomization temperatures were 1300 °C and 1800 °C, respectively. In the analysis of molybdenum, the use of calcium as a chemical modifier proved advantageous. The optimized temperatures were 1900 °C (pyrolysis) and 2800 °C (atomization).

The methods were applied to quantify manganese and molybdenum in extracts prepared from normal colored and discolored leaves of DP. The discolored leaves had higher levels of manganese and less molybdenum compared to the regular leaves. These results can be one explanation for the discoloration. Manganese toxicity manifests itself in the chlorosis of leaves. In addition, high levels of manganese are associated with a lower uptake of molybdenum. A deficiency of certain molybdenum-containing enzymes can also induce chlorosis.

In summary, with the aid of the developed GF AAS methods, light was shed on the connection between the two minerals, manganese and molybdenum, and the discoloration of the leaves. These findings could be used in the future to further optimize the cultivation of DP.

## Figures and Tables

**Table 1:** Time–temperature program for the quantification of manganese (λ = 279.4817 nm) in the leaf extracts of *Dioscorea polystachya*.

Operation	Temperature (°C)	Heating Rate (°C s^−1^)	Holding Time (s)	Argon Flow
Drying	90	10	10	Maximal
Drying	100	5	10	Maximal
Drying	120	5	15	Maximal
Pyrolysis	1300	150	15	Maximal
Auto-zero	1300	0	5	Stop
Atomization	1800	1500	5	Stop
Cleaning	2200	500	5	Maximal

**Table 2:** Time–temperature program for the quantification of molybdenum (λ = 313.2594 nm) in the leaf extracts of *Dioscorea polystachya*.

Operation	Temperature (°C)	Heating Rate (°C s^−1^)	Holding Time (s)	Argon Flow
Drying	90	10	10	Maximal
Drying	100	5	10	Maximal
Drying	120	5	15	Maximal
Pyrolysis	400	50	20	Maximal
Pyrolysis	1900	500	10	Maximal
Auto-zero	1900	0	5	Stop
Atomization	2800	1500	5	Stop
Cleaning	2850	500	5	Maximal

**Table 3:** Analytical figures of merit of the GF AAS method to quantify manganese in leaf extracts of *Dioscorea polystachya*.

Parameter	Value
Linear working range	2–20 µg L^−1^
Correlation coefficient (R^2^)	0.9995
Characteristic mass (m_0_)	0.67 ± 0.02 pg
Limit of detection (LOD)	0.67 µg L^−1^
Limit of quantification (LOQ)	1.83 µg L^−1^
Recovery/Precision (2 µg L^−1^)	98.5%/1.9%

**Table 4:** Analytical figures of merit of the GF AAS method to quantify molybdenum in leaf extracts of *Dioscorea polystachya*.

Parameter	Value
Linear working range	10–100 µg L^−1^
Correlation coefficient (R^2^)	0.9909
Characteristic mass (m_0_)	21.4 ± 3.4 pg
Limit of detection (LOD)	9.94 µg L^−1^
Limit of quantification (LOQ)	37.64 µg L^−1^
Recovery/Precision (40 µg L^−1^)	100.7%/3.8%

**Table 5:** Manganese and molybdenum content in the extracts of different normal colored and discolored leaves of *Dioscorea polystachya* expressed as mass of mineral per mass of leaves (mg kg^−1^).

Coloration of Leaves	Extract	Manganese Content (mg kg^−1^)	Mean (mg kg^−1^)	Molybdenum Content (mg kg^−1^)	Mean (mg kg^−1^)
Normal leaves	NL-1	39.09	30.85	26.59	18.41
	NL-2	45.25		12.35	
	NL-3	10.62		8.84	
	NL-4	28.46		25.84	
Discolored Leaves	DL-1	33.39	37.27	14.29	15.87
	DL-2	66.47		13.08	
	DL-3	35.48		20.77	
	DL-4	13.74		15.35	

## Data Availability

The data presented in this study are available in the manuscript.
